# Recognition of Lyso-Phospholipids by Human Natural Killer T Lymphocytes

**DOI:** 10.1371/journal.pbio.1000228

**Published:** 2009-10-27

**Authors:** Lisa M. Fox, Daryl G. Cox, Jennifer L. Lockridge, Xiaohua Wang, Xiuxu Chen, Louise Scharf, David L. Trott, Rachel M. Ndonye, Natacha Veerapen, Gurdyal S. Besra, Amy R. Howell, Mark E. Cook, Erin J. Adams, William H. Hildebrand, Jenny E. Gumperz

**Affiliations:** 1 Department of Medical Microbiology and Immunology, University of Wisconsin School of Medicine and Public Health, Madison, Wisconsin, United States of America; 2 Department of Microbiology and Immunology, University of Oklahoma Health Sciences Center, Oklahoma City, Oklahoma, United States of America; 3 Department of Biochemistry and Molecular Biology, University of Chicago, Chicago, Illinois, United States of America; 4 Department of Animal Science, University of Wisconsin, Madison, Wisconsin, United States of America; 5 Department of Chemistry, University of Connecticut, Storrs, Connecticut, United States of America; 6 School of Biosciences, University of Birmingham, Birmingham, United Kingdom; Weatherall Institute of Molecular Medicine, United Kingdom

## Abstract

Natural killer T (NKT) cells are a subset of T lymphocytes with potent immunoregulatory properties. Recognition of self-antigens presented by CD1d molecules is an important route of NKT cell activation; however, the molecular identity of specific autoantigens that stimulate human NKT cells remains unclear. Here, we have analyzed human NKT cell recognition of CD1d cellular ligands. The most clearly antigenic species was lyso-phosphatidylcholine (LPC). Diacylated phosphatidylcholine and lyso-phosphoglycerols differing in the chemistry of the head group stimulated only weak responses from human NKT cells. However, lyso-sphingomyelin, which shares the phosphocholine head group of LPC, also activated NKT cells. Antigen-presenting cells pulsed with LPC were capable of stimulating increased cytokine responses by NKT cell clones and by freshly isolated peripheral blood lymphocytes. These results demonstrate that human NKT cells recognize cholinated lyso-phospholipids as antigens presented by CD1d. Since these lyso-phospholipids serve as lipid messengers in normal physiological processes and are present at elevated levels during inflammatory responses, these findings point to a novel link between NKT cells and cellular signaling pathways that are associated with human disease pathophysiology.

## Abstract 2

By identifying the lipid LPC as an endogenous antigen, recognized by the invariant subset of human NKT cells, this study establishes a novel link between these immunoregulatory cells and an inflammatory lipid mediator.

## Author Summary

A central tenet of immunology is that cellular responses that protect us from pathogens result from molecular recognition of foreign compounds (antigens). The role of self-antigens in immune activation is less clear. We show here that an endogenous lipid called lyso-phosphatidylcholine (LPC) is recognized as an antigen by a subpopulation of human T lymphocytes, called natural killer T (NKT) cells, and specifically by the best-studied subgroup of these cells known as invariant NKT (iNKT) cells. NKT cells have attracted the interest of immunologists because they can potently influence the outcome of diverse immune responses; for example, they can promote bacterial clearance and tumor rejection, and they can also quell autoimmune disease pathology. Previous studies indicated that NKT cells are activated by self-antigens, but the identity of the relevant compounds remained unclear. Our finding that LPC is a self-antigen for iNKT cells suggests that these lymphocytes are attuned to highly conserved lipid signaling pathways that are fundamental to normal physiological processes and are markedly up-regulated during inflammation. Thus, these results provide a new molecular basis for understanding how iNKT cells contribute to a wide variety of immune responses.

## Introduction

Natural killer T (NKT) cells are a unique subpopulation of T lymphocytes that display innate-like characteristics and can potently modulate adaptive immune responses [1],[2]. They are among the first cells to respond during microbial infections and produce a wide variety of cytokines that have multiple effects on other immune cells [3],[4]. NKT cells are characterized by a restricted T cell receptor (TCR) usage in which the TCRα chain is invariant, and the TCRβ chains show more limited variability than those of classical T lymphocytes. The T cell receptors of NKT cells are specific for a nonclassical antigen-presenting molecule called CD1d that presents lipids and glycolipids. One of the most remarkable features of NKT cells is the source of the antigens they recognize. Unlike classical MHC-restricted T cells, which are selected for recognition of non–self compounds, NKT cells have been found to recognize both self and foreign molecules [2],[3]. Thus, NKT cells become activated in vivo even when there is no external challenge, and this property may underlie many of their immunoregulatory effects as well as their rapid activation during infection [2],[5].

Based on their restricted TCR usage, it has been proposed that NKT cells recognize a conserved set of antigens. Consistent with this, NKT cells have been found to share recognition of a class of microbial lipids in which a galactose sugar is attached in an α-anomeric configuration to a sphingolipid or a diacylglycerol [6]–[8]. Recognition of this type of glycolipid appears to be conferred by an evolutionarily conserved antigen recognition “hotspot” within the T cell receptors of NKT cells [9]–[11]. It remains unclear whether the part of the TCR that varies from NKT cell to NKT cell confers additional individual antigen recognition properties; however, a number of reports have documented antigen-specific responses that are confined to subsets of the NKT cell population, suggesting that this may indeed be the case [12]–[14].

The molecular identity of the self-antigens responsible for endogenously activating NKT cells, and how these antigens stimulate beneficial immune functions rather than uncontrolled autoreactive pathology, are major unresolved mysteries. A series of studies has indicated that the self-antigens recognized by murine NKT cells are loaded into CD1d molecules within intracellular endosomal vesicles and require specialized processing steps that take place at these sites. Mutated CD1d molecules that do not traffic through the endosomal vesicular system fail to stimulate CD1d-dependent autoreactive responses by murine NKT cells and are not able to positively select NKT cells in vivo [15],[16]. Additionally, murine NKT cells show reduced responses to self-antigens if normal endosomal functioning is inhibited, for example, by the addition of pH-altering drugs or when lysosome-resident enzymes are genetically deficient [17],[18]. A glycolipid called isoglobotrihexosyl ceramide (iGb3) that is generated in lysosomal compartments through glycosidic cleavage of the mature tetra-glycosylated form has been identified as a self-antigen recognized by murine NKT cells [19]. However, this glycolipid is not required for the development and function of murine NKT cells in vivo, suggesting that other as yet unidentified compounds also function as NKT cell self-antigens [20].

In contrast to the murine system, the self-antigen responses of human NKT cells do not require lysosomal processes [21],[22]. Mutated human CD1d molecules that do not traffic through the endosomal system stimulated normal autoreactive responses by human NKT cell clones, and drugs that alter lysosomal pH also had no deleterious effect [22]. Similarly, antigen-presenting cells (APCs) that are genetically deficient in lysosomal lipid transfer proteins stimulated normal self-antigen responses by human NKT cells [21]. Moreover, although the iGb3 glycolipid is antigenic for a fraction of the human NKT cell subpopulation [19],[23], it is not clear that this is a self-antigen for human NKT cells, since current data suggest that the iGb3 molecule is not produced in humans due to the lack of functional genes for galactosyl transferase enzymes that are required for its biosynthesis [24]. These data, demonstrating disparity between the human and murine systems, suggest there may be significant differences in the nature of the self-antigens that regulate human and murine NKT cell responses. This potentially clinically important point will not be clarified until there is a molecular understanding of the CD1d ligands recognized by NKT cells of each species. Here, we have analyzed the responses of human NKT cells to lipids found within the ligand pool of secreted human CD1d molecules.

## Results

To identify self-antigens recognized by human NKT cells, we tested their responses to synthetic preparations of compounds that were identified in a pool of ligands eluted from human CD1d molecules [25]. Lipids were pulsed onto plate-bound recombinant human CD1d-Fc fusion protein and tested for their ability to stimulate cytokine secretion by a panel of human NKT cell clones. We have found from previous analyses that the CD1d-Fc fusion protein, which is produced in a hamster cell line, does not stimulate significant responses from our NKT cells unless an antigenic lipid is added [23]. Hence, because there is little or no detectable reactivity to CD1d ligands that may be endogenously present in the recombinant molecules, this assay provides a means of assessing NKT cell responses to added ligands, even if they are relatively weak agonists [26].

### Recognition of LPC

**Figure 1:**
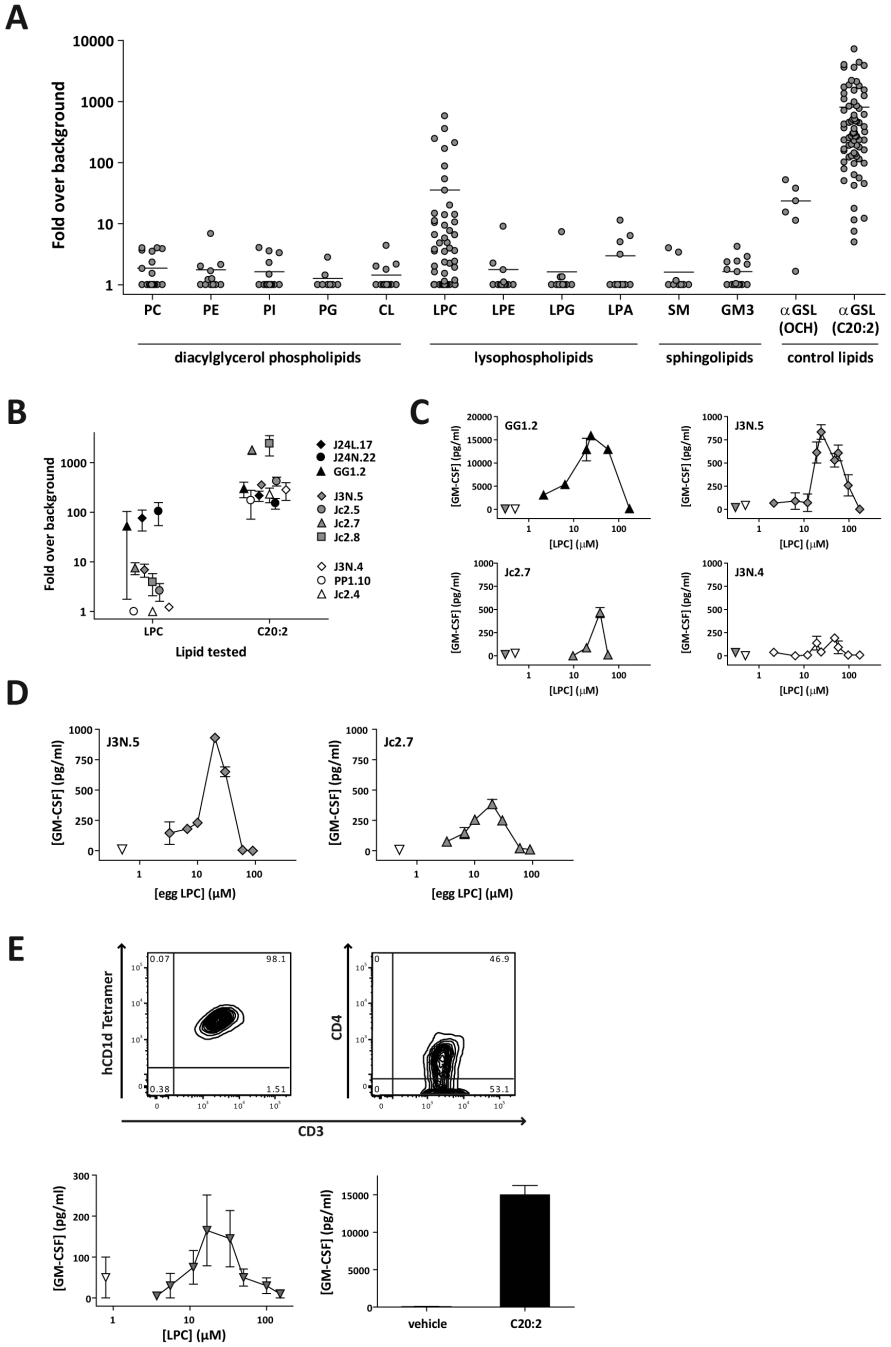
NKT cell recognition of LPC. (A) Human NKT cell clones were tested for cytokine secretion in response to immobilized recombinant CD1d molecules that were pulsed with lipid species that were found among the ligands eluted from human CD1d molecules [25]. Two α-galactosylsphingolipids (α-GSL OCH and C20:2) were tested in parallel as controls. Each dot represents an individual NKT cell clone's response to the indicated lipid, normalized by its response to vehicle alone. The results are compiled from analyses of 12 NKT cell clones. The control lipid α-GSL C20:2 was used at 0.6–1.2 µM; all other lipids were tested at concentrations of 20–75 µM. The horizontal line indicates the mean of the responses to each lipid. (B) Clonal variation in NKT cell responses to LPC. Ten different NKT cell clones were tested as described above for responses to LPC or a positive control antigen (C20:2 α-GSL). The plot shows the mean and standard deviation from two to four independent analyses of each clone, with the data expressed as the fold increase in cytokine secretion stimulated by lipid-pulsed CD1d as compared to vehicle-treated CD1d. (C) Cytokine secretion by four different NKT cell clones in response to CD1d molecules pulsed with the indicated concentrations of a synthetic preparation of C18:1 LPC. Inverted triangles show the cytokine secretion by each clone in the absence of recombinant CD1d molecules (shaded triangles), or to recombinant CD1d molecules treated with vehicle alone (open triangles). Results shown are from one representative experiment for each clone; similar dose-response curves were observed for each clone in two to four independent assays. (D) Dose-response curves for NKT cell clones to CD1d molecules pulsed with a preparation of LPC purified from chicken eggs. Similar results were observed in four independent assays. (E) Polyclonal NKT cells were isolated from human blood by magnetic sorting of cells labeled with a CD1d-Fc fusion protein loaded with the C20:2 α-GSL, and expanded in culture for 3–4 wk. Top panels show flow cytometric analysis of the expanded cells using α-GSL–loaded CD1d tetramer, anti-CD3, and anti-CD4 antibodies. Lower panels show cytokine secretion by the expanded NKT cells in response to plate-bound CD1d molecules pulsed with LPC, C20:2 α-GSL, or vehicle alone. Error bars in (B–E) indicate the standard deviation (SD). CL, cardiolipin; GM3, monosialoganglioside GM3; GM-CSF, granulocyte macrophage colony-stimulating factor; LPA, lyso-phosphatidic acid; LPC, lyso-phosphatidylcholine; LPE, lyso-phosphatidylethanolamine; LPG, lyso-phosphatidylglycerol; PC, phosphatidylcholine; PE, phosphatidylethanolamine; PG, phosphatidylglycerol; PI, phosphatidylinositol; SM, sphingomyelin.

Figure 1A shows a summary of responses by human NKT cell clones to glycerophospholipids and sphingolipids found within a pool of lipid ligands eluted from human CD1d molecules [25]. Ligand species were selected so as to include representative diacylated phospholipids (phosphatidylcholine, PC; phosphatidylethanolamine, PE; phosphatidylinositol, PI; and phosphatidylglycerol, PG), a tetra-acylated cardiolipin (CL) species, monoacylated lyso-phospholipids (lyso-phosphatidylcholine, LPC; lyso-phosphatidylethanolamine, LPE; lyso-phosphatidylglycerol, LPG; and lyso-phosphatidic acid, LPA), and the two most abundant sphingolipids (sphingomyelin, SM; and the ganglioside GM3). As a positive control, the CD1d-Fc molecules were pulsed with a form of the prototypical NKT cell antigen α-galactosylceramide (α-GalCer) that contains a 20-carbon fatty acyl chain with two unsaturations (C20:2) and is known to load particularly well into recombinant CD1d molecules in solution [27]. Since natural ligands generally have been found to stimulate weaker responses from NKT cells than α-GalCer, as another control, we also assessed NKT cell responses to a truncated form of α-GalCer called “OCH” that has been shown to be a weaker agonist for human NKT cells [26],[28]. Of the species tested from the CD1d ligand pool, LPC elicited the strongest NKT cell responses (Figure 1A). The NKT cell responses to LPC were generally 10- to 100-fold less than their responses to the C20:2 analog of α-GalCer and appeared similar to those induced by OCH (Figure 1A), suggesting that LPC is a weak to moderate agonist.

### Clonal Differences in LPC Recognition

Variation in the strength of the responses to LPC appeared to be largely due to reactivity differences among the NKT clones. Individual NKT cell clones were quite reproducible in their responses to LPC; some clones consistently showed strong responses, some regularly showed moderate or weak responses, and some repeatedly showed little or no detectable response (Figure 1B). The strength of individual NKT clone responses to LPC did not correlate with their responses to the C20:2 analog of α-GalCer (Figure 1B), suggesting that the LPC reactivity differences were not simply due to differing activation thresholds. Titrating the concentration of LPC used to prepulse the CD1d-Fc fusion protein yielded similar dose-response curves for all of the NKT cell clones. Significant responses above background were observed at LPC pulse concentrations from about 10 to 100 µM, with a peak at about 25 µM (Figure 1C). Notably, NKT cell responses were consistently diminished or absent at higher LPC pulse concentrations (Figure 1C).

To confirm that the NKT cell responses were due to recognition of LPC and not to a contaminant in the synthetic preparations of this compound, we tested LPC purified from chicken eggs. Dose-response curves to this LPC preparation, comprising a mixture of LPC species differing in their hydrocarbon chain lengths and double bonds, were similar to those for the synthetic LPC (Figure 1D). Thus, both synthesized and natural LPC preparations were recognized by the NKT cell clones, whereas synthesized preparations of related phospholipids were not.

To further investigate NKT cell recognition of LPC, we sorted a polyclonal population of NKT cells from the peripheral blood of a healthy volunteer donor using fluorescently labeled α-GalCer–loaded CD1d tetramers and expanded the cells in vitro for a short time (less than 1 mo) by stimulating them with PHA and IL-2 in the presence of irradiated autologous mononuclear cells. The resulting population of cells showed uniformly positive staining using an anti-CD3 antibody and α-GalCer–loaded CD1d tetramer, and contained approximately equal fractions of CD4^+^ and CD4^−^ cells (Figure 1E, top panels). The expanded polyclonal NKT cells showed a detectable cytokine response to plate-bound CD1d-Fc molecules pulsed with LPC and also responded to the C20:2 analog of α-GalCer (Figure 1E, bottom panels).

### Analysis of Other Ligand Species

**Figure 2:**
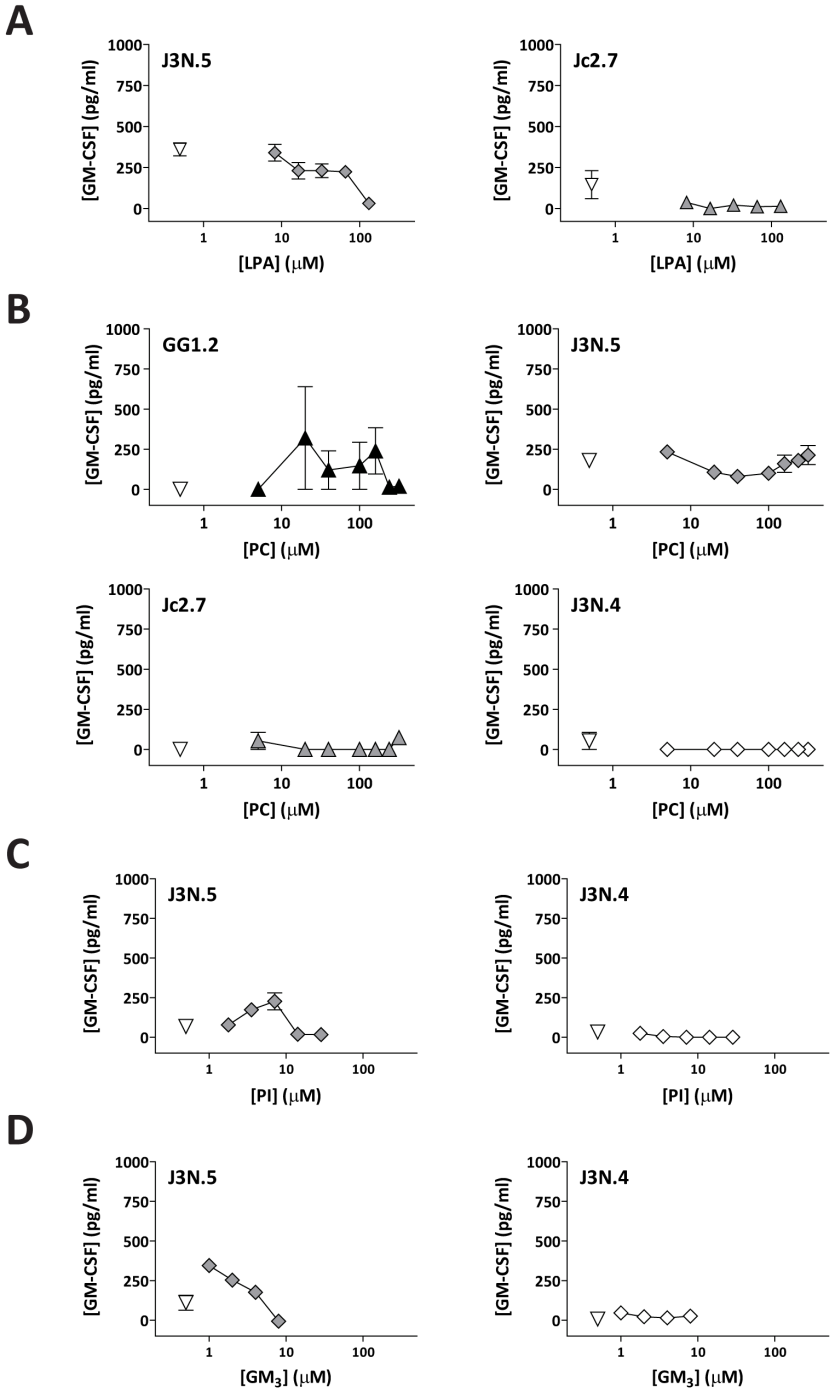
NKT cells show little or no response to other cellular lipids. (A) Representative examples of NKT cell clone responses to immobilized CD1d molecules pulsed with synthetic C18:1 LPA; (B) synthetic C18:1/C18:1 PC, (C) synthetic C18:1/C18:1 PI, and (D) purified ganglioside GM3. The plots show the means and standard deviations of three to four replicates.

Our screening of NKT cell lipid recognition showed occasional weak responses to other lipids identified within the CD1d ligand pool (Figure 1A). Therefore, we evaluated the NKT cell responses to these CD1d ligands using titrated doses of lipid. NKT cell clones that responded to LPC generally showed little or no recognition of other lyso-phospholipids, suggesting molecular specificity for LPC. For example, LPA, which is identical to LPC except for the absence of the choline head group, induced little or no NKT cell activation above background (Figure 2A). Diacylated PC sometimes stimulated very weak positive responses, but in most cases, there was no significant NKT cell activation from this lipid (Figure 2B), suggesting that the lyso- form contains antigenic features not present in the diacylated lipid.

We had previously identified a human NKT cell clone that consistently demonstrated specific responses to PI and PE, although other human NKT cell clones tested in parallel showed little or no recognition of these lipids [23]. In the current analysis, we found that PI was capable of eliciting weak responses from some NKT cell clones, but in general, this lipid failed to show stimulatory effects for the clones tested here (Figure 2C). PE only rarely elicited positive responses from the panel of NKT clones (Figure 1A, and unpublished data). We also failed to detect positive responses to plasmalogen forms of PE and PC (unpublished data). Weak but detectable NKT cell responses were sometimes observed to a purified preparation of the GM3 ganglioside, although in most cases, the results for this lipid were also negative (Figure 2D). Notably, a human NKT cell clone (J3N.4), from which we previously reproducibly observed positive responses to iGb3 [23], did not respond to the structurally related compound GM3 (Figure 2D). Sphingomyelin also generally stimulated no detectable response from the NKT cell clones (Figure 1A, and unpublished data). Thus, LPC was unique among the ligand species tested here in the strength, consistency, and dose-dependence of the NKT cell responses it elicited.

### Presentation of LPC by CD1d

**Figure 3:**
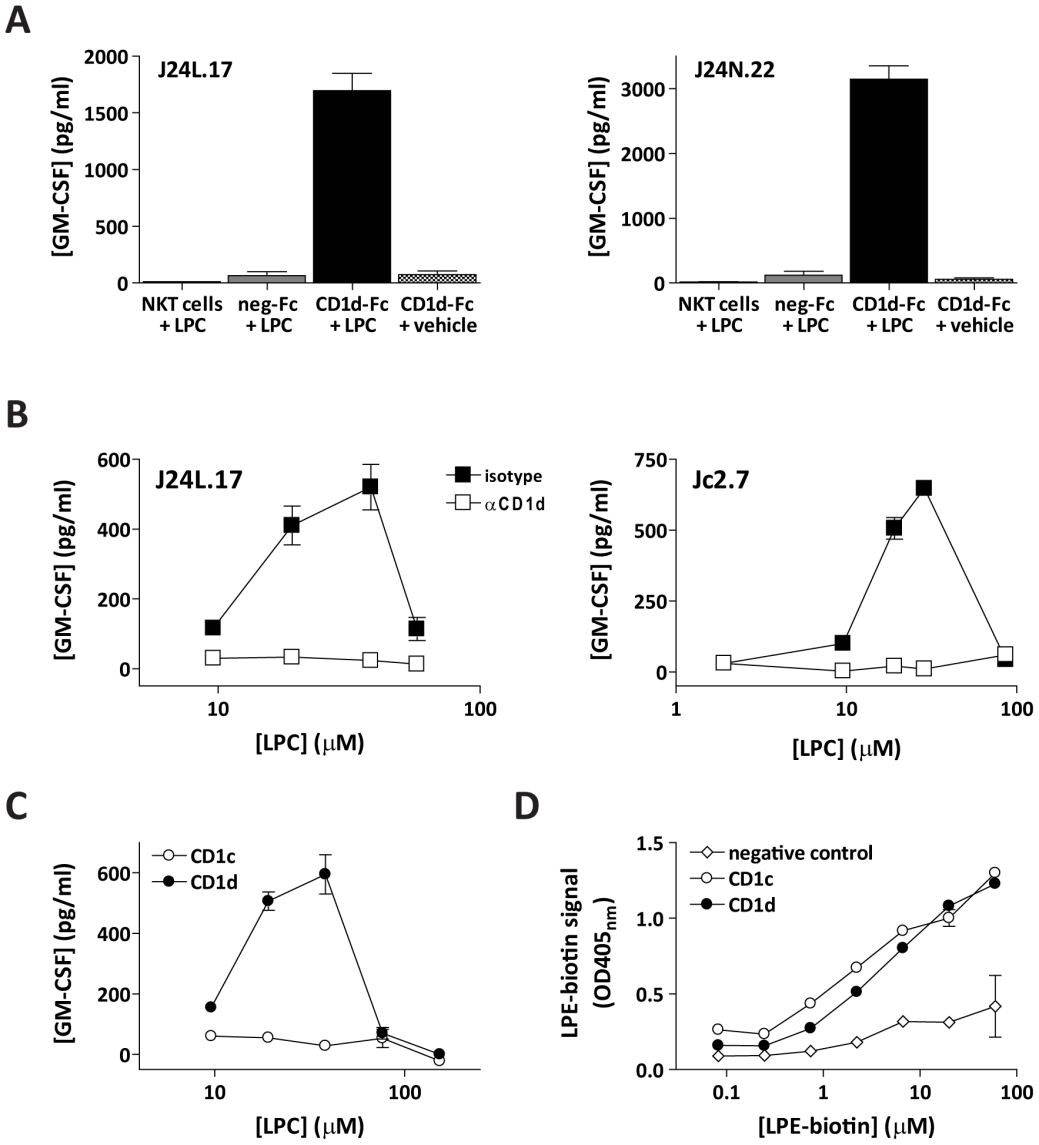
Responses to LPC are due to CD1d-mediated presentation. (A) NKT cell clones J24L.17 and J24N.22 were incubated directly with 20 µM of LPC (far left), with immobilized negative control antibody pulsed with 20 µM of LPC (second from left), immobilized CD1d-Fc molecules pulsed with 20 µM of LPC (second from right), or with immobilized CD1d-Fc pulsed with vehicle (far right), and cytokine secretion was quantitated by ELISA. Similar results were observed in four independent assays. (B) Responses of NKT cell clones JC2.7 and J24L.17 to CD1d molecules pulsed with LPC were blocked by inclusion of an anti-CD1d antibody but not an isotype-matched negative control antibody. Similar results were observed in two independent assays. (C) NKT cells respond to LPC-pulsed CD1d molecules, but not to the related isoform CD1c. The plot shows one representative experiment out of two, using clone J3N.5. Similar results were observed with three other NKT cell clones. (D) Biotinylated C18:1 LPE binds similarly to CD1d and CD1c molecules. Similar results were observed in three independent experiments.

Lyso-phospholipids are known to be highly bioactive molecules that can signal through G-protein–coupled receptors; therefore, it was possible that the responses we observed might be due to direct stimulation of NKT cells, rather than via TCR-mediated antigen recognition. To address this possibility, we performed a number of controls to confirm that the observed NKT cell responses were due to recognition of LPC in the context of CD1d. NKT cells that were incubated directly with LPC in the absence of CD1d molecules showed no detectable cytokine secretion, and similarly, there was no response to plate-bound negative control antibody that was prepulsed with LPC (Figure 3A). NKT cell responses to LPC-pulsed CD1d-Fc molecules were specifically blocked by an anti-CD1d antibody (Figure 3B). Additionally, the NKT cells did not respond to CD1c-Fc molecules that were prepulsed with LPC (Figure 3C), although we found that CD1c-Fc and CD1d-Fc molecules showed similar binding of a biotinylated lyso-phospholipid (Figure 3D). Together, these results demonstrate that NKT cell responses to LPC require presentation by CD1d molecules.

### Molecular Specificity of Recognition

**Figure 4:**
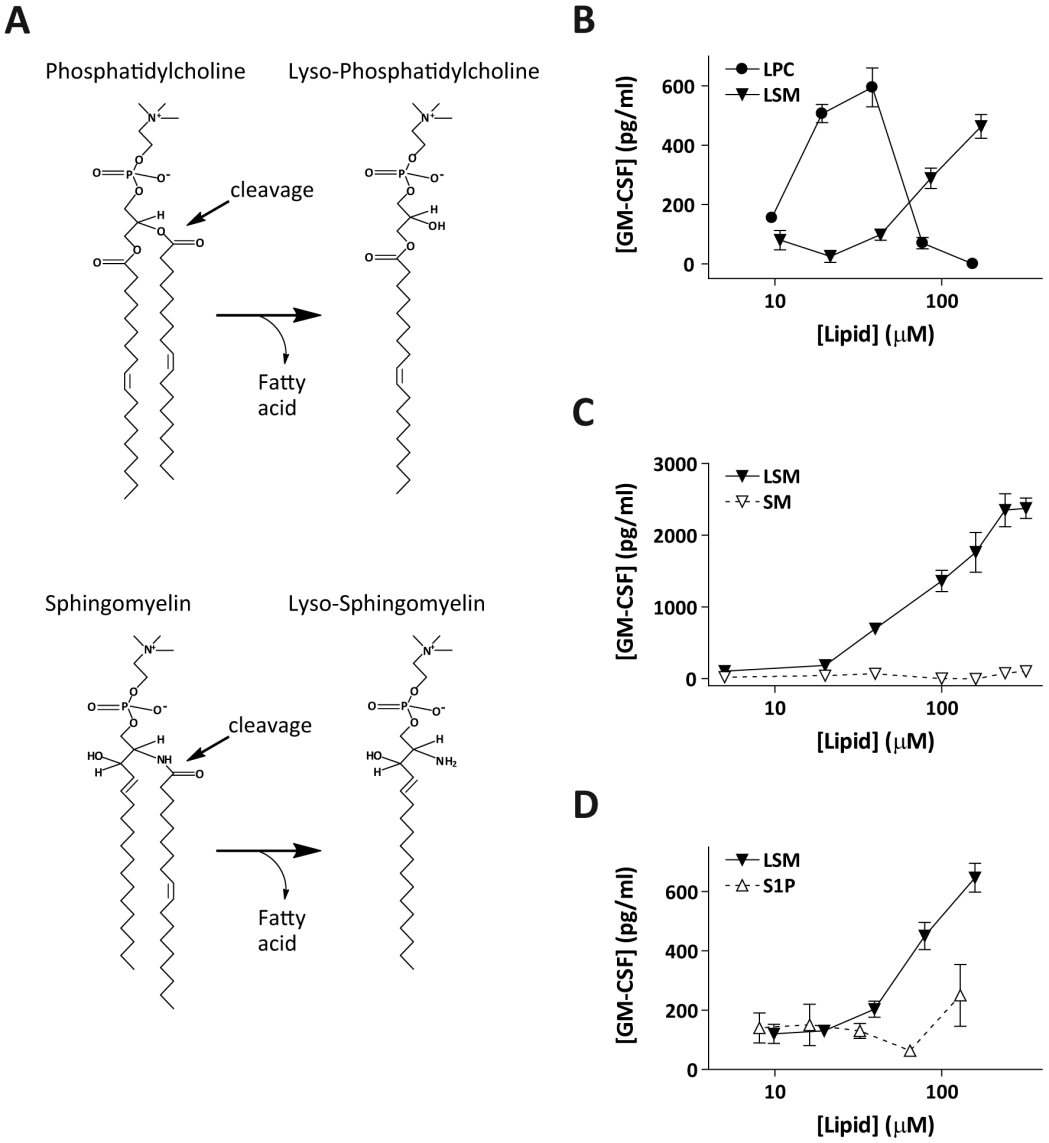
Molecular specificity of NKT cell recognition. (A) Chemical structures of di-C18:1 PC and SM, and their lyso derivatives LPC and LSM. (B) Response of NKT cell clone J3N.5 to CD1d molecules pulsed with the indicated concentrations of LPC or LSM. One representative experiment out of five is shown. Similar results were observed with two other NKT cell clones. (C) Only the lyso- form of sphingomyelin appears markedly antigenic for NKT cells. The results shown are from one representative experiment out of two using clone J3N.5. Similar results were observed with two other NKT cell clones. (D) Sphingosine-1-phosphate (S1P), which is identical to LSM except that it lacks the choline head group, was not recognized. Results shown are from one representative experiment out of three using clone Jc2.7. Similar results were observed with two other NKT cell clones.

In our initial screening of lyso-phospholipids found in a pool of eluted CD1d ligands [25], LPC was the only species that consistently stimulated cytokine secretion from most of the NKT cell clones (Figure 1A). Since the lipid tails of all of the lyso-phospholipids tested in this analysis were identical (i.e., C18:1), this suggests that NKT cell recognition is dependent on chemical features of the head group. To investigate this further, we tested the ability of LPC-reactive NKT cells to respond to lyso-sphingomyelin (LSM), a structurally related compound that was not found in the CD1d ligand pool. LPC and LSM can be generated by similar enzymatic cleavage of the diacylated phospholipids phosphatidylcholine (PC) and sphingomyelin (SM), resulting in removal of the fatty acyl chain and the generation of lyso- species that contain a choline head group linked by a phosphate ester to a single hydrocarbon tail (Figure 4A). NKT cell clones that recognized LPC consistently also showed responses to CD1d-Fc molecules that were prepulsed with LSM, although higher molar concentrations of LSM were required to stimulate responses (Figure 4B). In contrast, there was typically no detectable response to SM, the diacylated form (Figure 4C). Sphingosine 1-phosphate, a lyso- species lacking the choline head group, stimulated little or no response from the NKT cells (Figure 4D). Hence, NKT cell responses were specific for lyso-phospholipids containing a choline head group.

### Effect of Abundant Ligands on CD1d Antigen Loading

**Figure 5:**
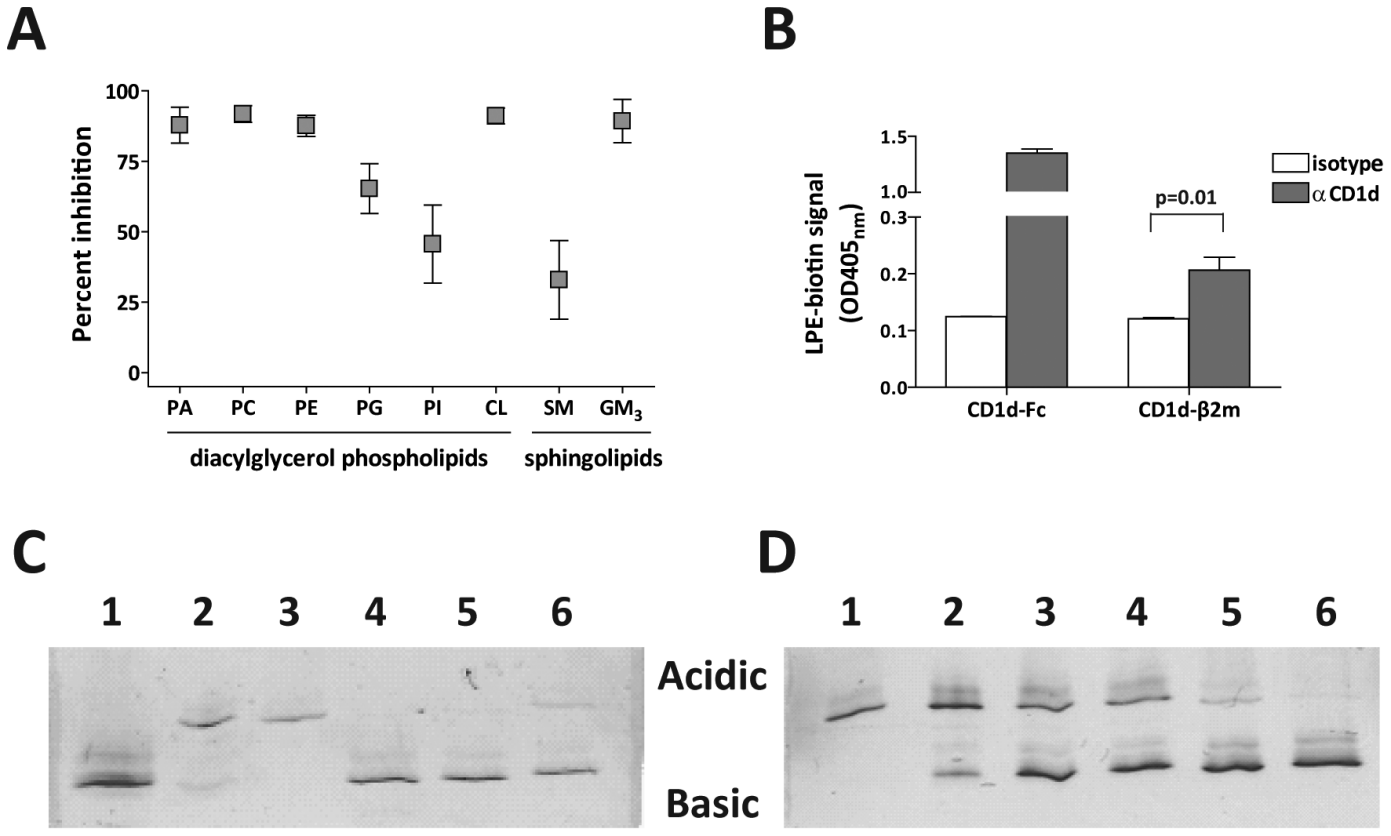
Effect of bound lipids on CD1d antigen loading. (A) Immobilized CD1d molecules were preincubated with the indicated lipids or treated with vehicle alone for 24 h. Unbound lipids were washed away, and the CD1d molecules were incubated with the α-GSL C20:2 for 24 h, then tested for the ability to stimulate NKT cell cytokine secretion. Percent inhibition was calculated by comparing NKT cell cytokine secretion in response to C20:2 pulsed onto CD1d molecules pretreated with lipid compared to CD1d pretreated with vehicle alone. The plot shows means and standard deviations of results compiled from seven independent experiments. (B) Biotinylated LPE (15 µM) was incubated with CD1d-Fc fusion protein or with secreted CD1d-β2m heterodimers that contain a mixture of bound ligands [25]. The samples were then incubated on plates coated with an anti-CD1d mAb (filled bars) or with an isotype-matched negative control mAb (open bars), and bound LPE was detected using a streptavidin-enzyme conjugate. OD450_nm_, optical density at 450 nm. (C) Native isoelectric focusing (IEF) analysis of lyso-phospholipid binding to recombinant CD1d molecules. Purified native CD1d molecules were mock-treated (lane 1), or preloaded with the trisialoganglioside GT1b (lanes 2–6), then incubated in solution with free lipids and separated by electrophoresis according to charge. Lane 3 shows GT1b-CD1d incubated with additional GT1b; lane 4 shows GT1b-CD1d incubated with a 4.5-fold molar excess of α-GalCer; lane 5 shows GT1b-CD1d incubated with a 3-fold molar excess of LPC; and lane 6 shows GT1b-CD1d incubated with a 3-fold molar excess of LPE. (D) Titration of the amount of lyso-phospholipid required to displace bound GT1b. Lane 1 shows GT1b-CD1d incubated in buffer with no LPE; lanes 2–6 show GT1b-CD1d incubated with the following molar ratios of LPE: lane 2 = 1∶1, lane 3 = 1∶2, lane 4 = 1∶3, lane 5 = 1∶5, and lane 6 = 1∶9.

Whereas lipids that appear to be abundant cellular ligands of human CD1d, such as SM, PC, PE, PI, CL, or GM3 [25],[29],[30], showed little or no antigenicity in this analysis, it is nevertheless possible that they play an important role in the CD1d antigen-presenting system by modulating the ability of other more antigenic lipids to load into CD1d molecules. We therefore investigated whether binding of these lipids to CD1d could block the subsequent presentation of an antigenic glycolipid. Recombinant CD1d-Fc molecules were preincubated with diacylated phospholipids or sphingolipids. The recombinant CD1d-Fc was then washed and incubated with a saturating concentration of the C20:2 analog of α-GalCer and tested for the ability to stimulate cytokine secretion by NKT cell clones. Pretreatment with several of the lipids, including PA, PC, PE, CL, and GM3, consistently resulted in almost complete blocking of the response to the C20:2 antigen (Figure 5A). In contrast, pretreatment with PG, PI, or SM resulted in only partial blocking of C20:2 (Figure 5A). These results suggest that a fraction of the CD1d molecules exiting the secretory pathway (e.g., those containing PG, PI, or SM) may be receptive to binding extracellular diacylated lipids such as C20:2 at the cell surface.

We next investigated the ability of lyso-phospholipids to bind to CD1d molecules containing cellular ligands. We have found that we can readily detect specific association of biotinylated LPE with recombinant CD1d-Fc molecules (Figure 3D). However, it is not clear whether the CD1d-Fc fusion proteins used in these experiments contain endogenous lipids, and if they do, whether these modulate the binding of exogenously added lipids. Therefore, we investigated the binding of biotinylated LPE to purified CD1d-β2m heterodimers that were produced in a human lymphoblastoid cell line, and for which the bound ligands have recently been characterized as a mixture of phospholipids and sphingolipids [25]. Although the signal was much lower than that observed for the CD1d-Fc fusion protein, the purified CD1d-β2m molecules also yielded a biotin signal that was significantly above the background, indicating the presence of bound LPE (Figure 5B). These results indicate that lyso-phospholipids can bind to CD1d molecules containing a complex mixture of cellular lipids.

To further investigate, we tested whether lyso-phospholipids can cause the dissociation of diacylated lipids from CD1d molecules. Recombinant human CD1d molecules produced in insect cells have a uniform charge distribution and can be visualized as a single major band on a native isoelectric focusing (IEF) gel (Figure 5C, lane 1). When the CD1d molecules are loaded with a charged lipid such as the trisialoganglioside GT1b, the band shifts due to the acidic charge of the bound lipid (Figure 5C, lanes 2 and 3). Binding of a neutral lipid (e.g., α-GalCer) to the CD1d-GT1b complex replaces the bound GT1b and is therefore associated with loss of the acidic charge (Figure 5C, lane 4). We found that addition of a 3-fold molar excess of either LPC or LPE to the CD1d-GT1b complex resulted in dissociation of 70%–80% of the bound GT1b, as assessed by the reduced intensity of the acidic band and the increased intensity of the basic band (Figure 5C, lanes 5 and 6). Titrating the concentration of lyso-phospholipid that was added to the CD1d-GT1b complex demonstrated that even a 1∶1 molar ratio of lyso-phospholipid to CD1d was sufficient to induce dissociation of approximately 30% of the bound GT1b (Figure 5D, lane 2), with nearly complete GT1b dissociation observed at molar ratios of 5∶1 or higher (Figure 5D, lanes 3–6). These results demonstrate that lyso-phospholipid loading into CD1d molecules is not prevented by previously bound diacylated lipids.

### Cell Surface Presentation of LPC

**Figure 6:**
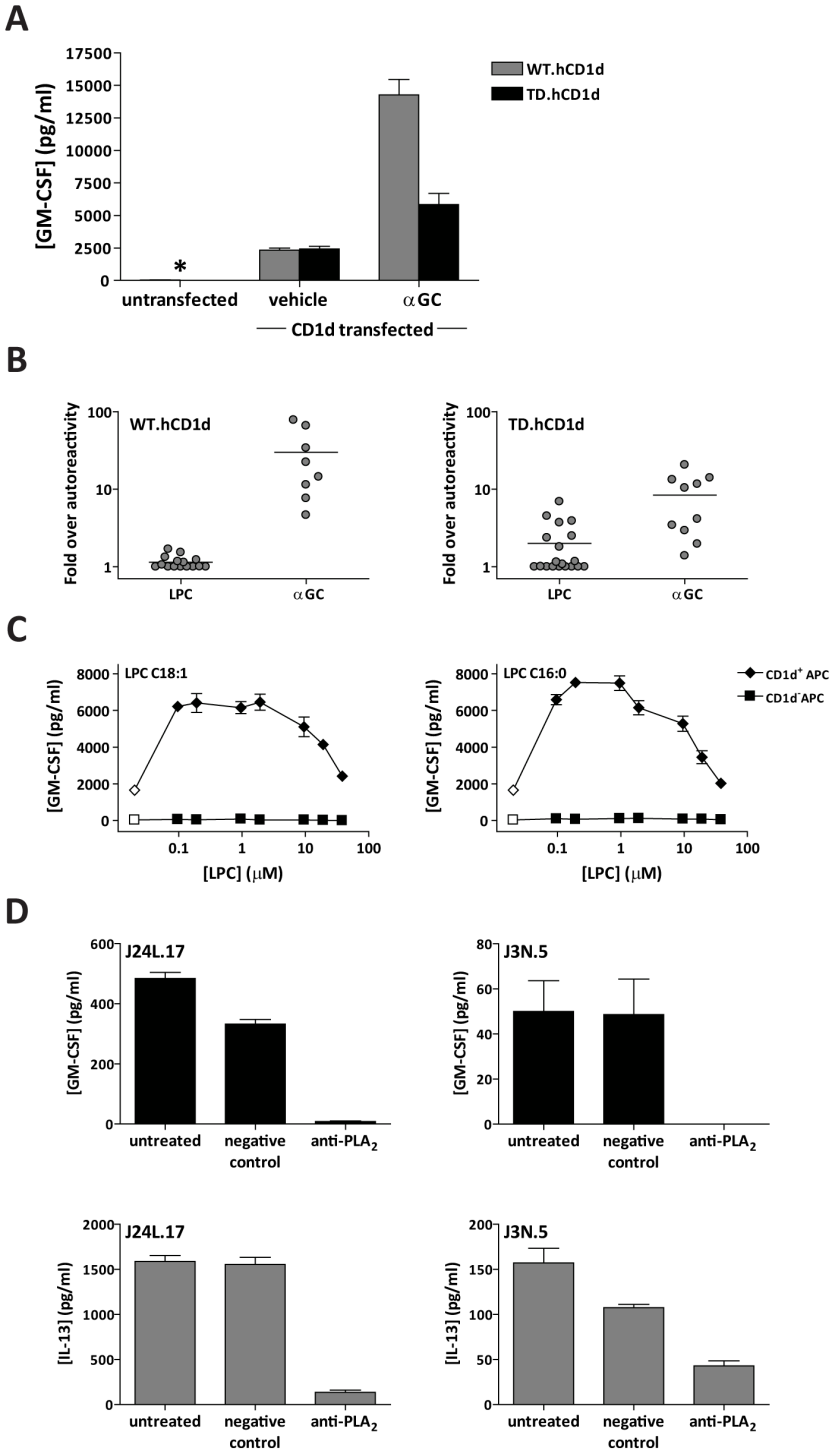
Presentation of LPC by cell surface CD1d. (A) Human lymphoblastoid cell lines transfected with either wild-type (WT) or cytoplasmic tail-deleted (TD) human CD1d molecules, or the untransfected parental cells (i.e., CD1d-negative) were tested for the ability to stimulate cytokine secretion by human NKT cell clones. The cells were pulsed with 7.5 nM α-GalCer (αGC) or mock treated, then washed and incubated with NKT cell clones. Culture supernatants were collected after 24 h and assayed for GM-CSF (a cytokine produced by the NKT cells) using a standardized ELISA. The plot shows one representative experiment out of three, using clone Jc2.7. Similar results were observed with three other NKT cell clones. The asterisk indicates signal that was below the limit of detection. (B) Transfectants expressing wild-type or tail-deleted CD1d were pulsed with 1–10 µM LPC or 7.5 nM α-GalCer, and used to stimulate human NKT cell clones. Each dot represents an independent analysis, with the data expressed as the amount of cytokine secreted in response to antigen-pulsed APCs normalized by the response to mock-treated APCs. The horizontal line indicates the mean of the responses. (C) Transfected cells expressing tail-deleted CD1d (CD1d^+^ APCs) or the untransfected parent cells (CD1d^−^ APCs) were pulsed with the indicated concentrations of C18:1 or C16:0 LPC, and used to stimulate clone Jc2.7. Similar concentration-dependent LPC responses were observed in three independent experiments. (D) Freshly isolated human monocytes were incubated for 24 h in culture medium (“untreated”), or in culture medium containing anti-sPLA_2_ IgY or negative control IgY, then washed and used to stimulate cytokine secretion by NKT cell clones J24L.17 and J3N.5. Culture supernatants were analyzed for GM-CSF and IL-13 concentration by ELISA; the plots show the means and standard deviations of triplicate samples. One representative experiment out of three is shown.

Previous studies have indicated that endosomal trafficking of CD1d is important for efficient presentation of certain exogenous lipids, such as α-GalCer, apparently because loading of α-GalCer into CD1d molecules occurs much more efficiently in endosomal vesicles [22],[31]. We therefore investigated the role of CD1d endosomal trafficking for presentation of exogenous LPC by APCs. As observed previously [22], human lymphoblastoid cell lines transfected with cytoplasmic tail-deleted CD1d molecules that lack the amino acid motif required for reinternalization from the cell surface show reduced α-GalCer–dependent NKT cell responses compared with transfectants expressing wild-type CD1d (Figure 6A). However, wild-type and tail-deleted CD1d transfectants stimulate similar CD1d-dependent autoreactive responses by NKT cells (Figure 6A), demonstrating that endosomal recycling of CD1d molecules is not required for presentation of antigenic cellular lipids. Addition of LPC to wild-type CD1d transfectants resulted in statistically significant increases in NKT cell cytokine secretion in three out of 17 experiments (Figure 6B). In these cases, the magnitude of the enhancement was low (a mean increase of 1.48-fold±0.234). However, addition of LPC to tail-deleted CD1d transfectants produced significantly enhanced NKT cell responses in seven out of 19 experiments, and in these cases, the magnitude of the effect was greater (mean increase of 3.67-fold±1.727). These experiments indicate that it is possible for extracellular LPC to compete with endogenous ligands and load into cell surface CD1d molecules, although this pathway does not appear to be highly reproducible. Additionally, these results suggest that endosomal recycling of CD1d molecules limits the presentation of extracellular LPC.

To further investigate, we compared two species of LPC. Most LPC species that have been identified as CD1d cellular ligands contain carbon chains with one or more double bonds [25],[29]; however, the most abundant species of LPC in extracellular fluids is often the fully saturated C16:0 carbon chain form. We found that NKT cell responses to APCs pulsed with C18:1 and C16:0 LPC appeared similar (Figure 6C), suggesting that both species can load into cell surface CD1d. Importantly, the NKT cell responses to LPC-treated APCs were completely CD1d-dependent, since the CD1d-negative parental cell line that was pulsed with LPC did not stimulate NKT cell cytokine secretion (Figure 6C). Interestingly, similar to our results using recombinant CD1d-Fc molecules for presentation, the LPC-dependent responses were highly concentration dependent and consistently appeared diminished or abrogated when the APCs were pulsed with high levels of LPC (Figure 6C).

We next tested the effect of blocking phospholipase A_2_ enzymes on the autoreactive responses of NKT cells. Human monocytes in peripheral blood constitutively express CD1d and stimulate CD1d-dependent cytokine secretion by human NKT cells in the absence of added antigens [32]–[34]. We isolated monocytes from human peripheral blood and preincubated them for 24 h with a polyclonal preparation of chicken antibodies (IgY) directed against secreted phospholipase A_2_ (sPLA_2_), or with a negative control preparation of polyclonal IgY [35]. The monocytes were then washed and used to stimulate cytokine secretion by human NKT cell clones. Monocytes that were pretreated with the anti-sPLA_2_ antibody showed significantly reduced stimulation of NKT cell cytokine secretion compared to those that were treated with the negative control antibody, or to untreated monocytes (Figure 6D). Importantly, monocyte cell surface expression of CD1d was not reduced by anti-sPLA_2_ antibody pretreatment (unpublished data). These results point to an important role for PLA_2_ enzymes, key producers of LPC in vivo, in the activation of NKT cells by physiological APCs.

### Responses to LPC by Human Peripheral Blood Lymphocytes

**Figure 7:**
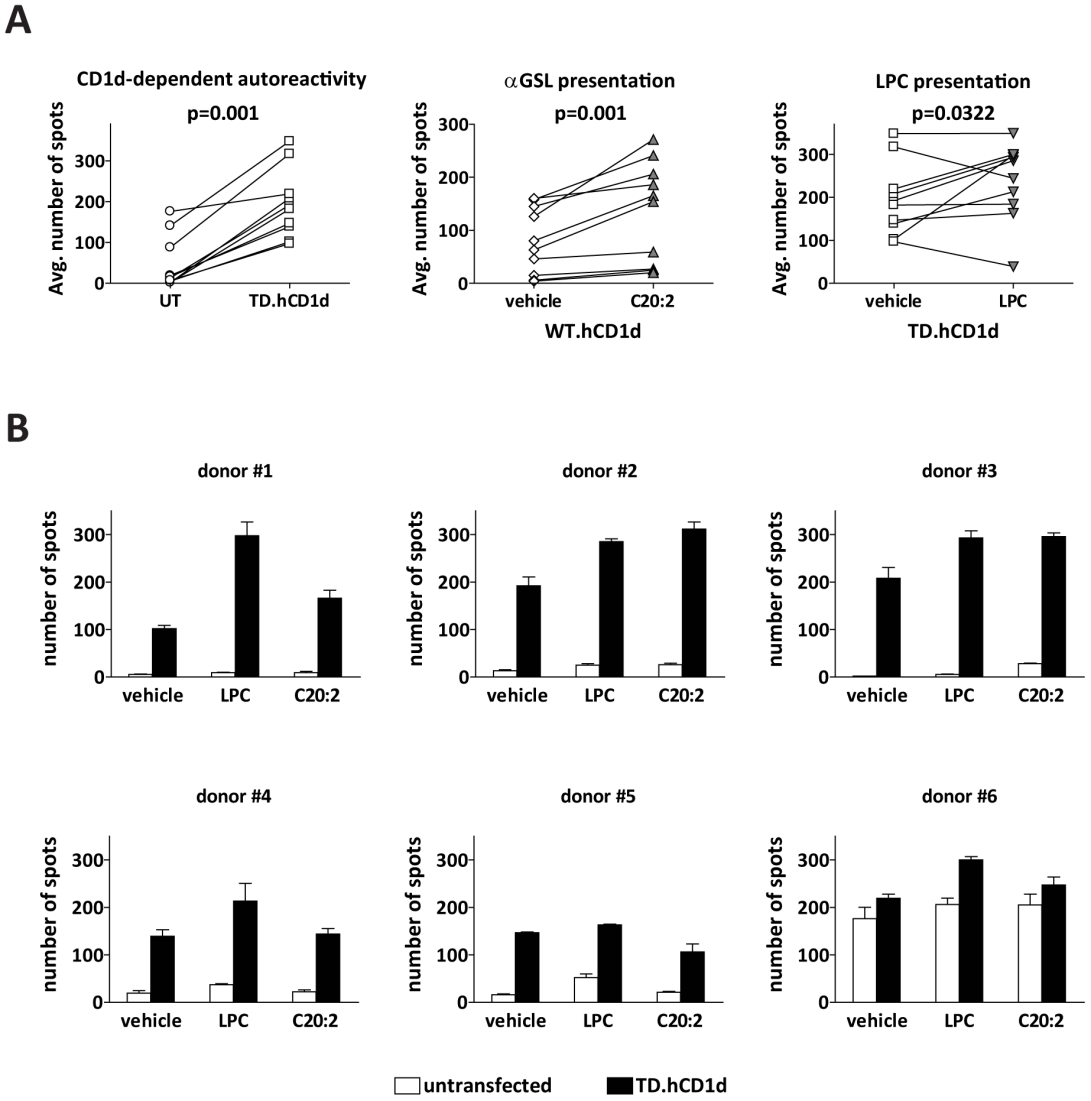
Activation of human peripheral blood lymphocytes. CD1d-transfected or untransfected APCs were pulsed with antigen or treated with vehicle alone, and then used to stimulate freshly isolated PBLs from ten healthy human donors. The frequency of IFNγ-secreting cells in each sample was assessed by ELISpot analysis. (A) Comparison of the average number of positive spots from triplicate wells of PBLs stimulated with the following APCs: left plot, untransfected (UT) APCs versus CD1d tail-deleted transfectant (TD.hCD1d) treated with vehicle alone; middle plot, wild-type CD1d transfectant (WT.hCD1d) treated with either vehicle or C20:2 α-GSL; right plot, tail-deleted CD1d transfectant treated with either vehicle or LPC. The data were statistically analyzed using a one-tailed Wilcoxon signed-rank *t*-test, yielding the *p*-values shown on the plots. (B) Results from individual donor samples that showed statistically significant increases in IFNγ spots in response to APCs treated with LPC. The plots show the mean numbers of spots detected from untransfected APCs (open bars) or tail-deleted CD1d transfectants (filled bars) that were prepulsed with the indicated lipid antigens or with vehicle alone. The *p*-values for responses to CD1d-transfected cells pulsed with LPC versus vehicle alone are as follows: donor #1 *p* = 0.0027; donor #2 *p* = 0.0096; donor #3 *p* = 0.0302; donor #4 *p* = 0.105; donor #5 *p* = 0.0021; and donor #6 *p* = 0.0021.

To further investigate the physiological role of LPC presentation by CD1d, we analyzed IFNγ responses by human peripheral blood lymphocytes (PBLs) directly ex vivo. Lymphocytes were freshly isolated from ten healthy adult donors, and tested by ELISpot analysis for cells that produced IFNγ in response to CD1d transfected or untransfected APCs. Because the APCs used for these experiments do not express MHC class II molecules on the cell surface and have reduced MHC class I expression [36], they should not stimulate marked alloreactive responses from the peripheral blood T cell populations of most donors. Consistent with this, most donors (seven out of ten) showed little or no IFNγ secretion (i.e., less than 20 spots per well) in response to the untransfected APCs (Figure 7A, left plot). However, PBL samples that were incubated with CD1d-transfected APCs consistently showed significantly increased numbers of spots (Figure 7A, left plot), suggesting that exposure to APCs expressing CD1d stimulated lymphocytes within the samples. Notably, the increased IFNγ production did not require the CD1d^+^ APCs to be prepulsed with antigen, suggesting that the responses are due to recognition of an endogenous antigen. PBL samples that were incubated with CD1d-transfected APCs prepulsed with C20:2 consistently showed a further increase in the number of spots (Figure 7A, middle plot), suggesting that additional T cells were activated by CD1d-mediated presentation of the α-GalCer analog. Most donors (eight out of ten) showed increased numbers of spots in response to CD1d tail-deleted APCs pulsed with LPC, compared to CD1d tail-deleted APCs treated with vehicle alone (Figure 7A, right plot). Six of the eight “responding” donors showed marked increases in the number of spots detected in response to the LPC-pulsed APCs (Figure 7B). These data suggest that CD1d-restricted T cells that respond to LPC as an antigen are present in the blood of healthy human adults.

## Discussion

The results presented here show that a fraction of human NKT cells specifically recognize LPC and LSM. Recognition of these lipids was observed using NKT cells that express semi-invariant T cell receptors and recognize a class of foreign antigens called α-GSLs [23],[26]. Semi-invariant NKT cells (or “iNKT” cells) such as these have been associated with beneficial immunoregulatory effects in a variety of murine models and also appear deficient in certain human autoimmune conditions [1]–[3]. It has been hypothesized that iNKT cell recognition of self-antigens allows them to perform immunoregulatory functions without foreign antigenic stimulation; however, the specific mechanisms by which this may occur have remained unclear. Our results indicate that the functions of NKT cells may be regulated by conserved lipid signaling pathways that operate during normal physiology and that have elevated activity during pathophysiological processes.

It has recently been shown that LPC can be isolated from human CD1d molecules purified from human lymphoblastoid cell lines [25],[29], providing strong evidence that lyso-phospholipids such as LPC can successfully compete with other types of self-lipids for loading into CD1d molecules. LPC is produced by the action of PLA_2_ enzymes, which are a functionally defined superfamily comprising at least 15 distinct types of proteins that localize to a variety of intracellular and extracellular sites [37]. Therefore, multiple sources of LPC may be available for loading into CD1d molecules. For example, stimulation of APCs by growth factors, cytokines, neurotransmitters, hormones, and other extracellular signals can lead to the activation of cytoplasmic PLA_2_ enzymes and release of LPC into the cytoplasm [38]. Additionally, the recent identification of a lysosome-resident PLA_2_ enzyme that is up-regulated in human monocytic cells upon stimulation through the retinoid X receptor suggests that LPC is produced within lysosomes after certain kinds of cellular activation [39]. Finally, several types of secreted PLA_2_ enzymes produce LPC by cleaving PC on the outer leaflet of the plasma membrane [40], and this LPC could load into CD1d molecules at the cell surface.

Our data indicate that secreted PLA_2_ enzymes are important for autoantigenic stimulation of NKT cells, since treatment of monocytes with an IgY preparation that was raised against purified sPLA_2_ protein specifically blocked their subsequent activation of NKT cells. This finding is consistent with the possibility that the cell surface is an important site of LPC production for loading into CD1d molecules. However, it is not clear from our results that high concentrations of extracellular LPC facilitate the activation of iNKT cells, since we have consistently found that NKT cells show little response to CD1d-mediated presentation of LPC when the lipid is added in concentrations above about 50 µM. The reason for this is unknown; our binding studies suggest that LPC does bind to CD1d molecules at these lipid concentrations. Nevertheless, this failure of high concentrations of LPC to activate NKT cells may be physiologically significant, since it occurs when either plate-bound recombinant CD1d-Fc molecules or CD1d-transfected APCs are used for LPC presentation. We also find that transfectants expressing wild-type CD1d molecules (which continuously recycle from the cell surface through endosomal compartments and back to the cell surface) show only a limited ability to present exogenously added LPC, whereas transfectants expressing tail-deleted CD1d molecules that are deficient in internalization from the cell surface appear more efficiently able to present exogenous LPC. This observation suggests that the normal recycling of CD1d molecules on APCs may limit the presentation of extracellular LPC. Thus, it remains to be determined whether autoreactive iNKT cell activation is most effective when LPC is produced at concentrations and cellular locations that are associated with normal physiological states or is further enhanced by elevated extracellular levels of LPC that are associated with inflammation.

We show here that lymphocytes that produce IFNγ in response to CD1d^+^ APCs are consistently present in the peripheral blood of healthy adult humans, and that for many donors, there is an increase in the frequency of IFNγ-producing cells when the APCs are prepulsed with LPC. It is not clear whether the LPC-reactive lymphocytes detected in this analysis are iNKT cells or whether they belong to a different subset of CD1d-restricted T cells. For example, blood samples from human multiple myeloma patients were recently reported to contain elevated frequencies of LPC-reactive CD1d-restricted T cells [41]. However, the LPC-reactive T cells from multiple myeloma patients did not utilize the characteristic T cell receptor of NKT cells and demonstrated skewed cytokine production, suggesting that they comprise a distinct CD1d-restricted T cell population [41]. Since LPC accumulates to greatly increased concentrations in blood and other bodily fluids in chronic inflammatory conditions such as multiple myeloma, it is possible that the T cell populations detected in blood of multiple myeloma patients were specifically expanded as a result of the disease state. It is not clear whether these LPC-reactive T cells play a pathogenic or a regulatory role in multiple myeloma.

Unfortunately, it has been difficult for us to gauge peripheral blood frequencies of LPC-reactive T cells in healthy donors because we have not obtained reproducible staining using LPC-loaded CD1d tetramers. Thus, it is not clear what fraction of the total iNKT cell population normally recognizes LPC, or what fraction of the total LPC-reactive T cell population is normally comprised of iNKT cells. However, our results do clearly demonstrate that not all iNKT cells recognize LPC. Approximately 75% of the NKT cell clones tested (eight out of 12) showed responses to LPC, whereas the remainder did not respond to this antigen but did respond well to the α-GSL used as a control. The ability of individual NKT cell clones to respond to LPC was generally very reproducible, and therefore, the most likely explanation for the clonal variation is that the TCR β-chain sequences of some clones permit recognition of this antigen, whereas other TCR β-chain sequences do not. Since the NKT cell clones that failed to respond to LPC nevertheless demonstrate detectable autoreactive responses to CD1d molecules expressed on APCs ([23], and unpublished data), these results suggest that some iNKT cells may recognize another, as yet unidentified, endogenous ligand. Alternatively, our results are also consistent with the possibility that additional autoreactive responses by iNKT cells result from recognition of very weak agonists that are abundant constituents of the ligand pools of human CD1d molecules, such as diacylated glycerophospholipids (e.g., PC, PI, and PE) and glycosphingolipids such as GM3 [25],[29].

It has recently been demonstrated that an autoreactive subset of noninvariant CD1d-restricted T cells found in mice can recognize sulfatide, a glycolipid derived from myelin, and that a lyso- form of sulfatide is more potently antigenic than the diacylated form [42],[43]. Hence, it may be a common finding that lyso-lipid species are more antigenic for CD1d-restricted T cells than their diacylated counterparts. Thus, perhaps autoreactive CD1d-restricted T cells monitor endogenous levels of cleaved lipids. In this case, oxidizing agents and lipases that generate these compounds may play a key role in the activation of these natural T cell populations. This possibility adds a new dimension to observations that sPLA_2_ enzymes play important roles, not only in inflammatory conditions, but also in host defense during microbial infections [40], since part of the immunological effects of these enzymes may result from their production of antigens that stimulate CD1d-restricted T cells. Similarly, the observation that lyso-phospholipids such as LPC can serve as potent immune adjuvants that enhance antigen-specific antibody production and cytotoxic T cell activation raises the possibility that these effects of LPC may involve the specific activation of NKT cells [44], since NKT cells are known to potently enhance memory responses by antigen-specific B cells and T cells [45],[46]. Thus, understanding the role of self-antigens such as LPC in regulating the responses of human NKT cells and other CD1d-restricted T cell populations may provide critical new insights into beneficial immune activation as well as disease pathology.

## Materials and Methods

### Lipid Antigens

The glycosphingolipid α-GalCer and its OCH and C20:2 structural analogs were synthesized as described previously [27],[47]. Synthetic preparations of the following lipids were obtained commercially (Matreya or Avanti Polar Lipids): C18:1/C18:1 phosphatidic acid, C18:1/C18:1 phosphatidylcholine, C18:1/C18:1 phosphatidylethanolamine, C18:1/C18:1 phosphatidylglycerol, C18:1/C18:1 phosphatidylinositol, C18:1/C18:1/C18:1/C18:1 cardiolipin, sphingomyelin (containing a C18:1 acyl chain), C18:1 lyso-phosphatidic acid, C18:1 and C16:0 lyso-phosphatidylcholine, C18:1 lyso-phosphatidylethanolamine, C18:1 lyso-phosphatidylglycerol, lyso-sphingomyelin, and sphingosine-1-phosphate. Purified preparations of the ganglioside GM3 (from bovine buttermilk) and lyso-phosphatidylcholine (from chicken eggs) were purchased from Avanti Polar Lipids. Diacylated lipids were dissolved in DMSO at a concentration of 100 µg/ml and stored frozen at −20°C. Lyso-phospholipids were dissolved in 50% DMSO/dH_2_O at a concentration of 400 µg/ml and stored frozen at −20°C. Lipids were warmed to room temperature, then sonicated at 60°C in a heated water bath for 20 min before use.

### NKT Cells

Human NKT cell clones were established as described previously [23], and maintained at 37°C with 5% CO_2_ in the following culture medium: RPMI 1640; 2 mM l-glutamine; 100 µg/ml penicillin and streptomycin; 10% fetal bovine serum (Hyclone); 5% bovine calf serum (Hyclone); 3% human AB serum (Atlanta Biologicals); supplemented with 400 U/ml recombinant human IL-2 (Chiron). The NKT cell clones were periodically restimulated by incubating them with irradiated allogeneic peripheral blood mononuclear cells (PBMCs) and 30 ng/ml anti-CD3 monoclonal antibody (mAb) (clone SPVT-3b). Polyclonal NKT cells were expanded from freshly isolated PBMCs from a healthy adult donor as follows: monocytes and B lymphocytes were removed by magnetic depletion using anti-CD14 and anti-CD19 microbeads (Miltenyi Biotec), and the remaining cells were incubated with human CD1d tetramer loaded with the C20:2 analog of α-GalCer, then the labeled cells were separated using goat anti-mouse IgG magnetic microbeads (Miltenyi Biotec). The positively selected cells were stimulated to proliferate by exposure to irradiated autologous PBMCs, in medium containing 250 ng/ml PHA-p. Recombinant human IL-2 (Chiron) was added after 2 d at a concentration of 40 U/ml, and titrated up to 400 U/ml over a period of 10 d. Experiments were performed on the polyclonal NKT cells within 3–4 wk of the initial sorting from fresh blood.

### CD1d Transfectants

APCs expressing wild-type or tail-deleted CD1d molecules were generated using the human lymphoblastoid 3023 cell line, as described previously [22]. The untransfected 3023 parental cell line was maintained in the following culture medium: RPMI 1640; 2 mM l-glutamine; 100 µg/ml penicillin and streptomycin; 5% bovine calf serum (Hyclone); 5% fetal bovine serum (Hyclone); 0.5 mg/ml G418 (Mediatech). For the transfected cell lines, this culture medium was supplemented with 0.5 µg/ml puromycin (Sigma-Aldrich).

### Recombinant CD1d Antigen-Presenting Assay

Recombinant human CD1d-Fc fusion protein was prepared as previously described [23]. CD1d-Fc fusion protein and anti-CD11a antibody (clone HI111, BioLegend) were coated onto high protein binding 96-well microtiter plates at 0.5 µg and 0.05 µg per well, respectively. Where indicated, the CD1d-Fc was replaced by human CD1c-Fc fusion protein or an isotype-matched negative control antibody (clone UPC-10). The wells were then incubated for 16–20 h at 37°C with C20:2, OCH, or test lipids diluted in 25% DMSO/dH_2_O. The wells were washed with sterile PBS, then RPMI, then RPMI containing 10% fetal bovine serum, and NKT cell clones (5×10^4^/well) were added in a final volume of 200 µl/well in culture medium (RPMI 1640; 2 mM l-glutamine; 100 µg/ml penicillin and streptomycin; 10% fetal bovine serum (Hyclone); 1 mM sodium pyruvate; 55 µM 2-mercaptoethanol; and nonessential amino acids). Where indicated, anti-CD1d antibodies (clone CD1d42.1) or an isotype-matched negative control antibody (clone P3) were added to the wells at a final concentration of 10 µg/ml, prior to the addition of NKT cells. Supernatants were collected after 18–24 h, analyzed for granulocyte macrophage colony-stimulating factor (GM-CSF) by ELISA (BioLegend), and quantified by comparison to recombinant human GM-CSF standards (PeproTech). Using this protocol, the means ± standard deviations of the background GM-CSF secretion were as follows: NKT cells exposed to plate-bound anti-CD11a without CD1d-Fc molecules, 47.9±88.9 (*n* = 44); NKT cells exposed to plate-bound anti-CD11a and untreated CD1d-Fc molecules, 44.2±67.9 (*n* = 69); NKT cells exposed to plate-bound anti-CD11a and vehicle-pulsed CD1d-Fc molecules, 45.2±80.2 (*n* = 210).

### Lyso-Phospholipid Binding to CD1 Molecules

Lyso-phosphatidylethanolamine (LPE) was biotinylated using Sulfo-NHS-biotin (Pierce), according to the manufacturer's protocol. The biotinylated lipid was dissolved in DMSO at a concentration of 100 µg/ml and sonicated at 60°C in a heated water bath for 20 min. Biotinylated LPE in PBS supplemented with 1 mg/ml BSA was incubated at the indicated concentrations for 2 h at 37°C with recombinant CD1c-Fc or CD1d-Fc fusion proteins, or with secreted native CD1d molecules produced in a human lymphoblastoid cell line as described [25]. The lipid-treated CD1 molecules were then incubated in microtiter plates coated with anti-CD1c mAb (clone F10/21A3), anti-CD1d mAb (clone CD1d42), or an isotype-matched negative control mAb (clone P3), to allow assessment of the CD1-dependent binding compared to the background, and biotinylated-LPE was detected using streptavidin-alkaline phosphatase (Zymed).

Lyso-phospholipid association with CD1d was also tested using an assay that measures displacement of a charged lipid ligand that is prebound to the CD1d [48]. A 6-His–tagged construct of the human CD1d ectodomain was coexpressed with human β_2_-microglobulin using a baculovirus insect expression system. CD1d protein was purified using Ni-NTA resin, followed by size-exclusion chromatography over a Superdex 200 column (GE Healthcare). The CD1d was loaded with purified trisialoganglioside GT1b (Matreya), as described previously [48]. Untreated or GT1b-loaded CD1d preparations were incubated for 2 h at 37°C at a protein concentration of 40 µM in HBS, in the presence of the indicated concentrations of α-GalCer, C18:1 LPC, C18:1 LPE, or GT1b as a control. The species were then separated according to charge on a native isoelectric focusing gel (IEF PhastGel, GE Healthcare), and protein bands were visualized by Coomassie stain.

### Effects of Prebound Lipids on Antigen Loading

To assess inhibitory effects on antigen presentation, microtiter plate wells coated with recombinant CD1d-Fc fusion protein and anti-LFA-1 mAb were incubated for 24 h at 37°C with vehicle (25% DMSO in dH_2_O), or with vehicle containing the indicated lipids at a final concentration of 75 µM. The wells were then washed with PBS, and a solution of 0.6 µM C20:2 (dissolved in PBS supplemented with 1 mg/ml BSA) was added and incubated for 24 h at 37°C. The wells were washed again, and NKT cell clones (5×10^4^/well) were added and incubated for 18–20 h at 37°C with 5% CO_2_. Supernatants were collected and analyzed by standardized ELISA for GM-CSF concentration. Percent inhibition was calculated by the following formula: 1−(GM-CSF produced in response to lipid pretreated CD1d/GM-CSF produced in response to vehicle pretreated CD1d)×100.

### Lipid Antigen Presentation by Cell Surface CD1d

Wild-type or tail-deleted CD1d transfected or untransfected 3023 human B lymphoblastoid cells were pulsed for 4 h at 37°C with α-GalCer, or lyso-phosphatidylcholine (LPC), or vehicle (DMSO) alone. The cells were washed with culture medium, then co-incubated at a 1∶1 ratio (5×10^4^/well each) with NKT cells, in a final volume of 200 µl. Supernatants were collected after 18–24 h, and analyzed for NKT cell production of GM-CSF using a standardized ELISA.

### PLA_2_ Blockade

Polyclonal anti-sPLA_2_ IgY antibodies were prepared by immunizing Single-Comb White Leghorn laying hens with sPLA_2_ IB enzyme purified from porcine pancreas (Novozyme) in complete Freund's adjuvant (CFA). Negative control IgY antibodies were prepared by immunizing the hens with CFA alone [35]. The two IgY antibody preparations were purified from egg yolks by extraction with polyethylene glycol, followed by dialysis using a 50 kDa molecular weight (MW) cutoff membrane. The presence of IgY specific for sPLA_2_ in the immunized antibody preparation was confirmed by ELISA, whereas the negative control antibody preparation showed no detectable anti-sPLA_2_ antibody signal (Figure S1A). The anti-PLA_2_ IgY preparation was capable of reducing the conversion of PC to LPC by a secreted PLA_2_ enzyme in vitro, whereas the negative control IgY did not have this effect (Figure S1B). Additionally, we observed specific binding of the anti-PLA_2_ IgY to the cell surface of freshly isolated human monocytes (Figure S1C), suggesting that antibodies within the preparation recognize human PLA_2_ molecules. To assess the effect of anti-sPLA_2_ antibody treatment on NKT cell activation, monocytes were isolated from human PBMC samples by magnetic sorting using CD14 microbeads (Miltenyi Biotec). The monocytes were incubated for 18–24 h at 37°C and 5% CO_2_ in culture medium containing 20 µg/ml anti-sPLA_2_ or negative control IgY, or in culture medium with no added antibodies. The monocytes were washed with fresh medium and then combined at a 1∶1 ratio with NKT cells (5×10^4^/well of each). Supernatants were collected after 24 h and analyzed by ELISA for the production of GM-CSF and IL-13 (BioLegend).

### ELISpot Analysis

Human PBMCs were purified from fresh blood obtained from healthy adult donors using Ficoll-Paque density gradient centrifugation (GE Health Sciences), and B cells, monocytic cells, and plasmacytoid DCs were removed by magnetic sorting using beads specific for CD19, CD14, and BDCA-4 (Miltenyi). CD1d transfected or untransfected 3023 cells were incubated for 2 h at 37°C in culture medium containing LPC (10 µM), or the C20:2 analog of α-GalCer (260 nM), or vehicle (DMSO) alone, then washed and resuspended in fresh medium. PBMCs and APCs were added in a 1∶1 ratio (100,000 cells per well total) in serum-free medium (CELLect medium, MP Biomedicals) to triplicate wells of 96-well PVDF membrane plates (Whatman) coated with anti-human IFNγ mAb (clone NIB42 from BioLegend). The cells were incubated for 48 h at 37°C and 5% CO_2_. Secreted IFNγ was detected using biotinylated anti-human IFNγ mAb (clone M701B from Thermo Scientific), and revealed by development with streptavidin-alkaline phosphatase and BCIP/NBT chromogenic substrate. Spots were quantitated using AID 5.0 software. Background signal from analysis of PBMCs without added APCs was typically less than 20 spots per well.

## Supporting Information

Figure S1 **Specificity and function of anti-PLA_2_ IgY antibody.** Polyclonal anti-sPLA_2_ and negative control IgY antibodies were prepared by immunizing chickens with purified sPLA_2_ IB enzyme in complete Freund's adjuvant or with adjuvant alone. (A) Analysis of sPLA2 binding activity by ELISA. Microtiter plates were coated with purified porcine sPLA_2_ IB enzyme and blocked with bovine serum albumin. Dilutions of purified anti-PLA2 IgY or negative control IgY preparations were added to the wells, and bound IgY antibody was detected using HRP conjugated goat anti-IgY antibody. (B) Effect on sPLA_2_ enzyme activity. Synthetic C18:1/C18:1 phosphatidylcholine was incubated in aqueous solution with sPLA_2_ enzyme purified from bee venom in the presence of anti-sPLA_2_ or negative control IgY. Lipids were extracted using chloroform, then separated by thin layer chromatography (TLC) on silica gel 60 plates using chloroform-methanol-glacial acetic acid-water (90∶40∶12∶4 v/v) and visualized using iodine vapors. Synthetic preparations of LPC and PC were run in parallel to confirm the relative migration of the two species. The figure was made from a color photograph of a TLC plate that was converted to a black and white image. (C) Binding to human monocytes. Monocytes were isolated from peripheral blood of healthy human volunteer donors by magnetic sorting using anti-CD14 microbeads. The purified cells were incubated with 20 µg/ml anti-sPLA_2_ or negative control IgY, or with no primary antibody, then stained with a fluorescently labeled rabbit anti-IgY second-step antibody, and analyzed by flow cytometry.
(0.40 MB TIF)

## Abbreviations

APC: antigen-presenting cell
mAb: monoclonal antibody
NKT: natural killer T
PBL: peripheral blood lymphocyte
PBMC: peripheral blood mononuclear cell
