# Supplementary figures and images for: Recognition of Lyso-Phospholipids by Human Natural Killer T Lymphocytes
Source: PLoS Biol. 2009 Oct 27;7(10):e1000228. doi: 10.1371/journal.pbio.1000228 (PMC2760207; doi:10.1371/journal.pbio.1000228)

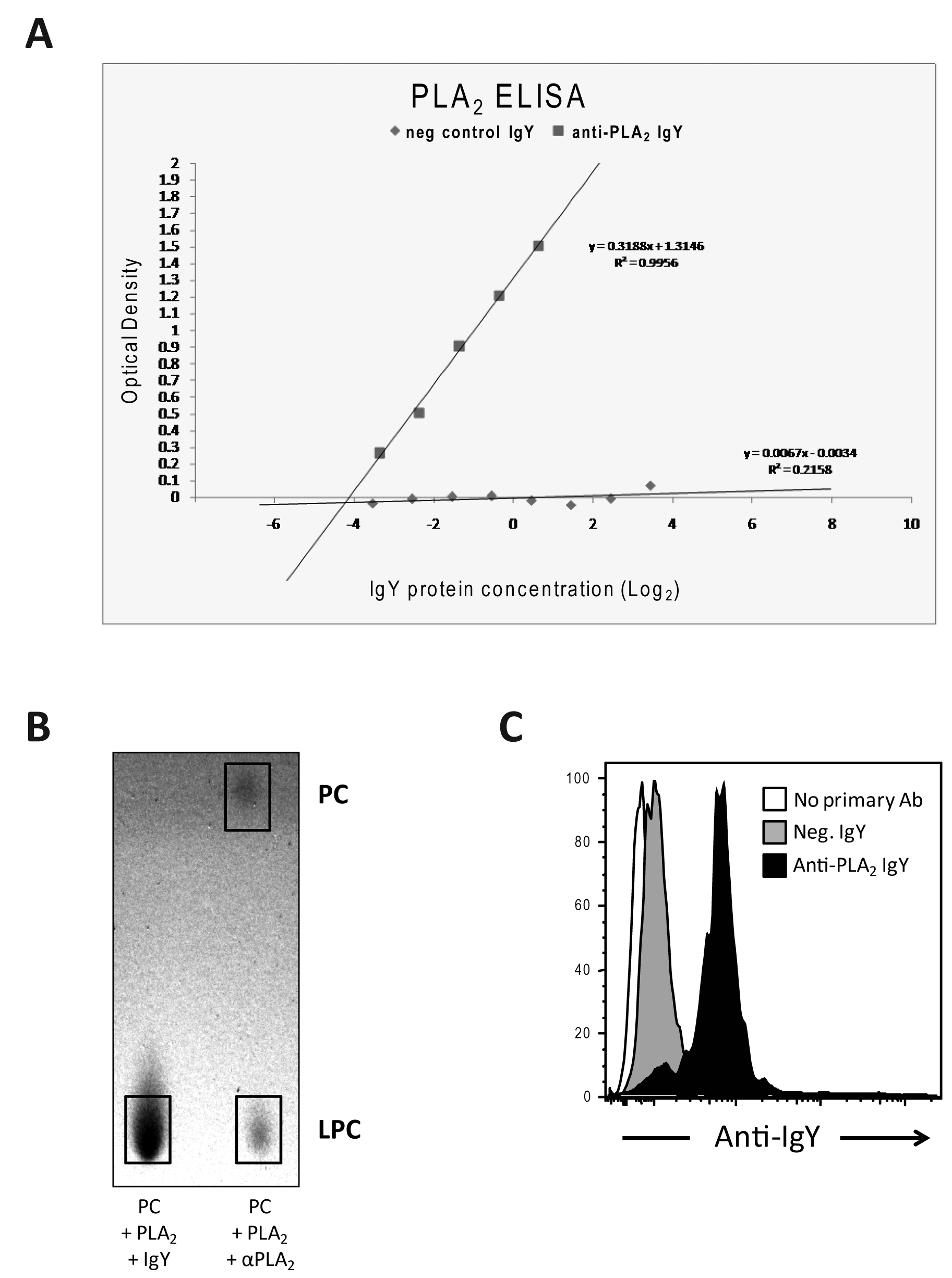

Supplement: Figure S1 — Specificity and function of anti-PLA2 IgY antibody. Polyclonal anti-sPLA2 and negative control IgY antibodies were prepared by immunizing chickens with purified sPLA2 IB enzyme in complete Freund's adjuvant or with adjuvant alone. (A) Analysis of sPLA2 binding activity by ELISA. Microtiter plates were coated with purified porcine sPLA2 IB enzyme and blocked with bovine serum albumin. Dilutions of purified anti-PLA2 IgY or negative control IgY preparations were added to the wells, and bound IgY antibody was detected using HRP conjugated goat anti-IgY antibody. (B) Effect on sPLA2 enzyme activity. Synthetic C18:1/C18:1 phosphatidylcholine was incubated in aqueous solution with sPLA2 enzyme purified from bee venom in the presence of anti-sPLA2 or negative control IgY. Lipids were extracted using chloroform, then separated by thin layer chromatography (TLC) on silica gel 60 plates using chloroform-methanol-glacial acetic acid-water (90∶40∶12∶4 v/v) and visualized using iodine vapors. Synthetic preparations of LPC and PC were run in parallel to confirm the relative migration of the two species. The figure was made from a color photograph of a TLC plate that was converted to a black and white image. (C) Binding to human monocytes. Monocytes were isolated from peripheral blood of healthy human volunteer donors by magnetic sorting using anti-CD14 microbeads. The purified cells were incubated with 20 µg/ml anti-sPLA2 or negative control IgY, or with no primary antibody, then stained with a fluorescently labeled rabbit anti-IgY second-step antibody, and analyzed by flow cytometry. (0.40 MB TIF) [file pbio.1000228.s001.tif]
